# Supplementary material for: Local magnetic moments in iron and nickel at ambient and Earth’s core conditions
Source: Nat Commun. 2017 Jul 12;8:16062. doi: 10.1038/ncomms16062 (PMC5510222; doi:10.1038/ncomms16062)
Supplement: Supplementary Information [file ncomms16062-s1.pdf]

File Name: Supplementary Information
Description: Supplementary Figures, Supplementary Tables, Supplementary Notes and Supplementary References

File Name: Peer Review File
Description:

SUPPLEMENTARY NOTE 1. THE COULOMB INTERACTION TENSOR

The general local Coulomb interaction Hamiltonian for a single five band impurity reads

$$\hat{H}_{\text{Full}} = \frac{1}{2}\sum_{ijkl} U_{ijkl}\hat{d}_i^\dagger \hat{d}_j^\dagger \hat{d}_l \hat{d}_k, \tag{1}$$

where $\hat{d}_i^\dagger, \hat{d}_i$ are the creation and annihilation operators for an electron in Wannier orbital $\phi_i(\mathbf{r})$ and the Coulomb interaction tensor is defined the "Slater" way [1]

$$U_{ijkl} = \int \mathrm{d}\mathbf{r}\ \mathrm{d}\mathbf{r}'\ \phi_i^*(\mathbf{r})\phi_j^*(\mathbf{r}')\ \frac{e^2}{|\mathbf{r}-\mathbf{r}'|}\ \phi_k(\mathbf{r})\phi_l(\mathbf{r}'). \tag{2}$$

We have estimated the Coulomb interaction tensor U_{ijkl} in a fully *ab-initio* way including the five $3d$- and the $4s$-orbital in the target space, by means of constrained-random phase approximation (cRPA)[2]. To avoid confusion over the definitions and values of the ubiquitous average parameters U and J we discuss them here carefully. One possible definition of the average parameters (for a five band system) is the following

$$U_{\text{K}} = \frac{1}{5}\sum_i U_{iiii}, \quad J_{\text{K}} = \frac{1}{20}\sum_{i\neq j} U_{iijj}. \tag{3}$$

Since this is in the spirit of Kanamori [3], we call these Kanamori averages and their values are reported in Supplementary Table 1. Another possible definition of average parameters is rooted in the Slater parametrization of the atomic Coulomb interaction [1, 4]

$$U_{ijkl} = \sum_{\alpha=0}^{4} a_\alpha(ik;jl)F^\alpha \tag{4}$$

and its density-density parts

$$U_{ijij} = U_{ij} = \sum_{\alpha=0}^{4} a_\alpha(ii;jj)F^\alpha, \quad U_{ijji}^{i\neq j} = J_{ij} = \sum_{\alpha=0}^{4} a_\alpha(ij;ji)F^\alpha, \tag{5}$$

where $a(ik;jl)$ is a shorthand for integrals of three spherical harmonics and can be expressed using Wigner $3j$-symbols, and F^α are the radial Slater integrals. Averaging over above expressions one obtains the "Slater" averages

$$U_{\text{S}} = \frac{1}{(2l+1)^2}\sum_{ij} U_{ijij} = F^0, \quad J_{\text{S}} = U_{\text{S}} - \frac{1}{2l(2l+1)}\sum_{ij}(U_{ijij} - U_{ijji}) = \frac{F^2+F^4}{14}, \tag{6}$$

relating back directly to the Slater integrals. Here $l = 2$ is the angular momentum of the d shell. The two averages are exactly related in the spherical (atomic) case by

$$U_{\text{K}} = U_{\text{S}} + \frac{8}{7}J_{\text{S}}, \quad J_{\text{K}} = \frac{5}{7}J_{\text{S}}, \tag{7}$$

which is still very well fulfilled for our cRPA matrix, see Supplementary Table 1.

For the calculations with Kanamori interaction we use the generalized Kanamori Hamiltonian

$$\hat{H}_{\text{Kanamori}} = \frac{1}{2}\sum_{i,j,\sigma} U_{ij}\hat{n}_{i,\sigma}\hat{n}_{j,-\sigma} + \frac{1}{2}\sum_{i\neq j,\sigma}(U_{ij}-J_{ij})\hat{n}_{i,\sigma}\hat{n}_{j,\sigma} + \frac{1}{2}\sum_{i\neq j,\sigma} J_{ij}\left(\hat{d}_{i,\sigma}^\dagger\hat{d}_{j,-\sigma}^\dagger\hat{d}_{i,-\sigma}\hat{d}_{j,\sigma} - \hat{d}_{i,\sigma}^\dagger\hat{d}_{i,-\sigma}^\dagger\hat{d}_{j,\sigma}\hat{d}_{j,-\sigma}\right), \tag{8}$$

while the density-density approximation consists only of the first two terms of above equation. The interaction matrices U_{ij} and J_{ij} are the density-density parts of the full cRPA matrix.

	Fe	Ni
$U_{iiii}(t_{2g})$	3.89	3.89
$U_{iiii}(e_g)$	3.80	4.05
U_{K}	3.85	3.95
J_{K}	0.72	0.78
U_{S}	2.71	2.72
J_{S}	1.00	1.08

SUPPLEMENTARY TABLE 1: Average values of the Coulomb interaction in eV using the two definitions given in the text as well as the diagonal matrix elements U_{iiii} for the t_{2g} and e_g orbitals respectively.

To better understand how the choice of interaction affects the desctiption of magnetism, we rewrite the atomic Coulomb-interaction Hamiltonian in terms of the total particle number operator $\hat{N}$, total spin $\hat{S}$ and pseudo total angular momentum $\hat{L}$. From the expression for density-density

$$\hat{H}_{\mathrm{dd}} = (U_{\mathrm{K}} - J_{\mathrm{K}}) \frac{\hat{N}(\hat{N}-1)}{2} - J_{\mathrm{K}} \left(\frac{\hat{N}^2}{4} + \hat{S}_z^2 - \hat{L}_z^2 \right), \tag{9}$$

and for Kanamori

$$\hat{H}_{\mathrm{Kana}} = (U_{\mathrm{K}} - 3J_{\mathrm{K}}) \frac{\hat{N}(\hat{N}-1)}{2} - 2J_{\mathrm{K}} \hat{\mathbf{S}}^2 - \frac{J_{\mathrm{K}}}{2} \hat{\mathbf{L}}^2 + \frac{5}{2} J_{\mathrm{K}} \hat{N} \tag{10}$$

one can see, that density-density tends to maximize $\hat{S}_z$, whereas Kanamori tends to maximize $\hat{\mathbf{S}}^2$. Thus density-density overestimates the magnetisation and Curie-temperature, which has been found in several model calculations [5, 6].

As shown in Fig. 1 a and b of the manuscript, the former overestimates T_{C} for both nickel and iron, as a consequence of the well-known absence of spin-flip processes [5, 6]. For nickel, both Kanamori and full Coulomb reproduce the experimental result with remarkable accuracy. α-iron (bcc structure) however has lower coordination than nickel and it is closer to half-filling, i.e. charge fluctuations and correlation effects are stronger. For these reasons, sizeable non-local corrections to DMFT are expected in iron [7]. In fact, even with the most sophisticated form of the Coulomb tensor, we find T_{C} about 30% larger than the experimental result [8–10].

SUPPLEMENTARY NOTE 2. INFLUENCE OF THE HUNDS COUPLING J

	$J_S = 0.8$eV	$J_S = 0.9$eV	J_S(cRPA)
Fe	1500K	1900K	2300K
Ni	530K	650K	860K

SUPPLEMENTARY TABLE 2: Estimates of T_C within the density-density approximation for different average Hunds couplings J_S.

The Hunds-rule coupling has a great influence on the critical temperatures in both iron and nickel. To explain the higher T_C we obtain, as compared to earlier work in Refs. [11–13] we performed density-density calculations varying the Hunds coupling. To this end we spherically averaged the Coulomb matrix from cRPA to be able to parametrize it simply via Slater integrals. In this way a change of the Hunds coupling is readily achieved. We employ values ranging from $J_S = \frac{1}{14}(F^2 + F^4) = 0.8$eV up to the cRPA estimate of 1.0eV for iron and 1.08eV for nickel. We see from Supplementary Table 2 that J has profound influence on the critical temperatures. A reduction of J_S to 0.9eV, the value used in the previous study by one of us [11], reduces the critical temperature by a few hundred Kelvins and gets us close to the result published in aforementioned paper. The remaining difference can most probably be traced back to the different value of U and the different DFT implementations and ways to construct the local Hamiltonian.

SUPPLEMENTARY NOTE 3. DOUBLE COUNTING CORRECTION

The Coulomb matrix elements calculated within the cRPA contain naturally the cubic symmetry of the crystal. This means, that unlike in the usual spherically symmetric parametrization of the Coulomb matrix the diagonal entries U_{iiii} are different for the t_{2g} and e_g orbitals. This poses a challenge to the so-called double counting correction of the DFT+DMFT formalism, which is usually estimated as an orbitally averaged Coulomb interaction [14]. The most widely used approaches are the fully-localized-limit (FLL) and around mean-field (AMF) corrections, giving for the double-counting potential μ_{DC} (using the Slater averages)

$$\mu_{DC}^{AMF} = U_S\left(N - \overline{n}\right) - J_S\left(\frac{N}{2} - \overline{n}\right) \tag{11}$$

$$\mu_{DC}^{FLL} = U_S\left(N - \frac{1}{2}\right) - J_S\left(\frac{N}{2} - \frac{1}{2}\right), \tag{12}$$

where N is the total number of $3d$ electrons and $\overline{n}$ is the occupation per orbital and spin. Since these depend on the filling of the shell N_d as well as the average Coulomb interaction U_S different corrections are in order when the Coulomb matrix is split into two different blocks.

A generalization of the AMF and FLL can be obtained following Ref. [14]. One arrives at the following orbitally dependent AMF and FLL corrections

$$\mu_{DC}^{AMF}(i) = n^0\left(\sum_j U_{ij} + \sum_{j,j\neq i}(U_{ij} - J_{ij})\right) \tag{13}$$

$$\mu_{DC}^{FLL}(i) = n^0\left(\sum_j U_{ij} + \sum_{j,j\neq i}(U_{ij} - J_{ij})\right) + \frac{1}{4}\left(n^0 - \frac{1}{2}\right)\left(\sum_j (U_{ij} - J_{ij})\right) \tag{14}$$

with the average DFT occupancy $n^0 = \frac{1}{2(2l+1)}\sum_{i,\sigma} n_{i,\sigma}$. Using either of these criteria gives results for the critical temperatures as well as the orbital occupancies close to the selfconsistency requirement on the charges from Supplementary Eq. 15 below.

Another possible approach is to constraint the total charge on the impurity, which is based on the Friedel sum rule [15]. The Friedel sum rule gives at zero temperature, a relationship between the extra states induced below the Fermi level by a scattering center (an impurity) and the phase shift at the chemical potential. For the Anderson model the extra states induced are given by the occupation number of the impurity states, and the scattering potential is the hybridization that affects the conduction electrons. We thus require the following to be true in self-consistency

		AMF(i)	FLL(i)	Tr(G)
Fe	$n_{t_{2g}}^{maj}$	0.84	0.85	0.86
	$n_{t_{2g}}^{min}$	0.57	0.57	0.56
	$n_{e_g}^{maj}$	0.86	0.87	0.89
	$n_{e_g}^{min}$	0.40	0.40	0.40
Ni	$n_{t_{2g}}^{maj}$	0.92	0.92	0.91
	$n_{t_{2g}}^{min}$	0.81	0.82	0.83
	$n_{e_g}^{maj}$	0.93	0.93	0.93
	$n_{e_g}^{min}$	0.86	0.87	0.88

SUPPLEMENTARY TABLE 3: Orbital occupancies for the different double counting corrections discussed in the text, within the density-density approximation. For iron we used $\beta = 6\text{eV}^{-1}$ and for Ni $\beta = 20\text{eV}^{-1}$.

$$\mathrm{Tr}\; G_{ij}^{\mathrm{imp}}(\beta) \stackrel{!}{=} \mathrm{Tr}\; G_{ij}^{0,\mathrm{imp}}(\beta). \tag{15}$$

The value of the imaginary time Green function at $\tau = \beta$ gives the orbital occupancy, thus the trace over them amounts to the total occupancy of the impurity. This works well in metallic systems [16], since in a metal the total particle number of the system and of the impurity are both very sensitive to small variations in the chemical potential μ and the double counting potential μ_{DC}. Also the likeness to the Friedel sum rule, that applies to metals, indicates that such a constraint will work for metals only. Since this constraint affects the chemical potential, μ, as well, both μ and μ_{DC} are adjusted self-consistently to fulfill it.

We have compiled orbital occupancies for the three different double counting corrections for one temperature close to T_{C} for Fe and Ni in Supplementary Table 3.

SUPPLEMENTARY NOTE 4. NI UNDER PRESSURE

In contrast to iron (see e.g. Ref. [17]), the bulk elastic properties of nickel are already well described within DFT [18–20]. To account for the effects of pressure in the case of Ni, we thus proceed as follows. First, we recorded energy vs. volume curves for Ni using the VASP code, employing nine different functionals as shown in Supplementary Table 4 and Supplementary Fig. 1. We used a $20 \times 20 \times 20$ point k-mesh centered at the Γ-point and a plane wave cutoff of 600eV. The resulting $E(V)$ curves are shown in Supplementary Fig. 1 a-c. Subsequently, these were fitted using the following equations of state (EOS):

- Murnaghan [21]

$$E(V) = E_0 + \frac{B_0 V}{B_0'}\left[\left(\frac{V_0}{V}\right)^{B_0'}\frac{1}{B_0'-1}+1\right] - \frac{B_0 V_0}{B_0'-1} \tag{16}$$

- Birch-Murnaghan [22]

$$E(V) = E_0 + \frac{9V_0B_0}{16}\left\{\left[\left(\frac{V_0}{V}\right)^{\frac{2}{3}}-1\right]^3 B_0' + \left[\left(\frac{V_0}{V}\right)^{\frac{2}{3}}-1\right]^2\left[6-4\left(\frac{V_0}{V}\right)^{\frac{2}{3}}\right]\right\} \tag{17}$$

- Poirer-Tarantola [23]

$$E(V) = E_0 + \frac{B_0V_0}{2}\left[\ln\left(\frac{V_0}{V}\right)\right]^2 + \frac{B_0V_0}{6}\left[\ln\left(\frac{V_0}{V}\right)\right]^3(B_0'-2) \tag{18}$$

- Vinet [24]

$$E(V) = E_0 + \frac{2B_0V_0}{(B_0'-1)^2}\left\{2-\left[5+3\left(\frac{V}{V_0}\right)^{\frac{1}{3}}(B_0'-1)-3B_0'\right]\exp\left[-\frac{3}{2}(B_0'-1)\left[\left(\frac{V}{V_0}\right)^{\frac{1}{3}}-1\right]\right]\right\} \tag{19}$$

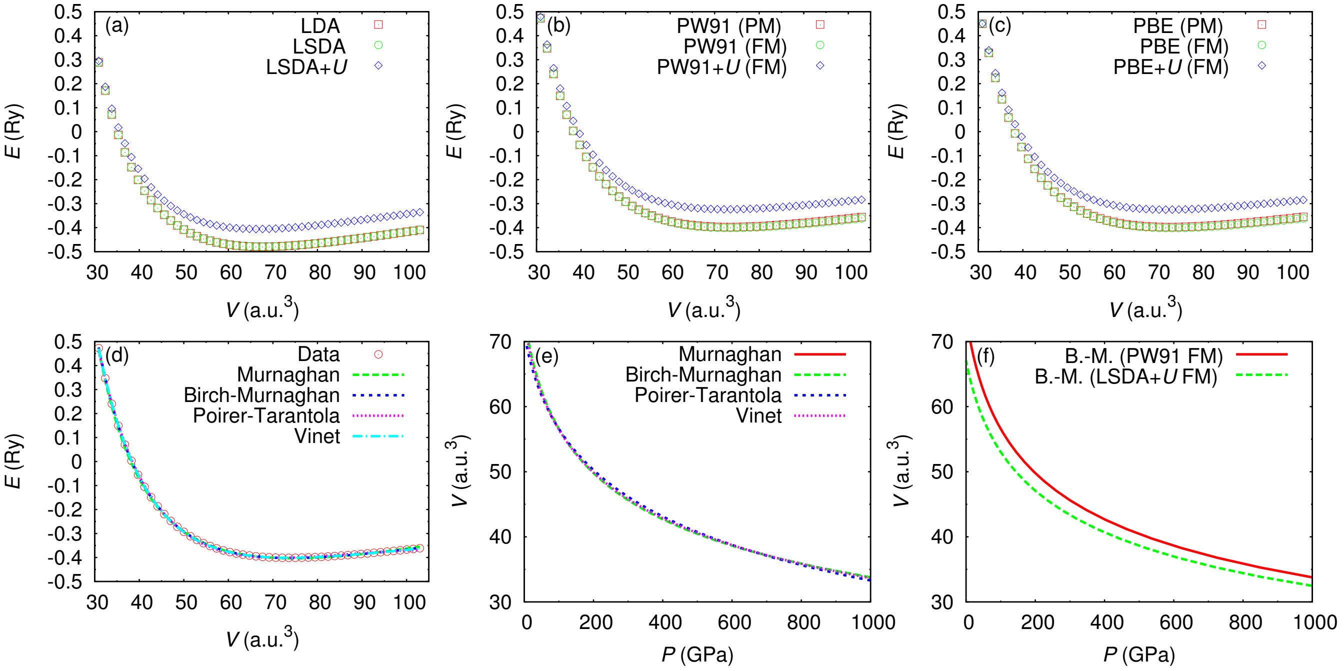

SUPPLEMENTARY FIG. 1: **Equation of state for Ni under pressure.**(a)-(c) Energy vs. Volume curves for all functionals used. PM indicates a paramagnetic calculation, FM a ferromagnetic one. (d) Comparison of the fitted equations of state using the PW91(FM) functional as an example (e) Comparison of the different equations of state for the $P(V)$ curve using the PW91(FM) functional as an example (f) Influence of the DFT functional on the $P(V)$ curve for the Birch-Murnaghan equation of state. The PW91 and the LSDA+U functionals constitute the extrema, all other functionals fall in between.

In above equations E_0 is the energy minimum, V_0 the volume at the minimum, B_0 is the bulk modulus and B_0' is its derivative with respect to pressure. The fits to the data are very good in all cases, as shown in Supplementary Fig. 1 d. The resulting fit parameters are compiled in Supplementary Table 4. Subsequently the obtained parameters were used to produce the $P(V)$ curves for each equation of state:

- Murnaghan

$$P(V) = \frac{B_0}{B_0'}\left[\left(\frac{V_0}{V}\right)^{B_0'} - 1\right] \tag{20}$$

- Birch-Murnaghan

$$P(V) = \frac{3B_0}{2}\left[\left(\frac{V_0}{V}\right)^{\frac{7}{3}} - \left(\frac{V_0}{V}\right)^{\frac{5}{3}}\right]\left\{1 + \frac{3}{4}(B_0' - 4)\left[\left(\frac{V_0}{V}\right)^{\frac{2}{3}} - 1\right]\right\} \tag{21}$$

- Poirer-Tarantola

$$P(V) = B_0\frac{V_0}{V}\left[\ln\left(\frac{V_0}{V}\right) + \frac{(B_0' - 2)}{2}\left[\ln\left(\frac{V_0}{V}\right)\right]^2\right] \tag{22}$$

- Vinet

$$P(V) = 3B_0\left(\frac{V}{V_0}\right)^{-\frac{2}{3}}\left[1 - \left(\frac{V}{V_0}\right)^{\frac{1}{3}}\right]\exp\left[-\frac{3}{2}\left(B_0' - 1\right)\left[\left(\frac{V}{V_0}\right)^{\frac{1}{3}} - 1\right]\right] \tag{23}$$

One set of such $P(V)$ curves is shown in Supplementary Fig. 1 e for the ferromagnetic PW91 calculation. Finally, we investigated the influence of the DFT functional on the $P(V)$ result. The two extremal curves in the case of the

EOS		LDA(P)	LSDA(F)	LSDA+U(F)	PBE(P)	PBE(F)	PBE+U(F)	PW91(P)	PW91(F)	PW91+U(F)
Murnaghan	E_0	-0.48	-0.48	-0.41	-0.40	-0.40	-0.33	-0.40	-0.40	-0.40
	V_0	68.90	68.90	68.10	74.04	74.50	73.67	74.33	74.72	74.50
	B_0	247.59	247.59	237.19	210.98	208.24	197.27	211.67	209.22	208.24
	B_0'	3.88	3.88	3.90	3.71	3.69	3.71	3.72	3.71	3.69
Birch-Murnaghan	E_0	-0.48	-0.48	-0.41	-0.40	-0.40	-0.33	-0.40	-0.40	-0.40
	V_0	67.89	67.89	67.08	73.26	73.73	72.84	73.53	73.95	73.73
	B_0	254.99	254.99	246.42	204.46	200.79	192.11	204.25	200.97	200.79
	B_0'	4.65	4.65	4.64	4.58	4.57	4.58	4.62	4.60	4.57
Poirer-Tarantola	E_0	-0.48	-0.48	-0.41	-0.40	-0.40	-0.32	-0.40	-0.40	-0.40
	V_0	66.02	66.02	65.35	70.81	71.26	70.42	70.98	71.39	71.26
	B_0	308.70	308.70	298.82	228.67	221.76	216.34	228.72	222.36	221.76
	B_0'	5.56	5.56	5.45	6.37	6.45	6.29	6.50	6.57	6.45
Vinet	E_0	-0.48	-0.48	-0.41	-0.40	-0.40	-0.32	-0.40	-0.40	-0.40
	V_0	67.17	67.17	66.38	72.61	73.09	72.17	72.88	73.31	73.09
	B_0	262.57	262.57	255.24	199.85	195.23	188.71	198.89	194.74	195.23
	B_0'	5.18	5.18	5.13	5.30	5.30	5.28	5.36	5.36	5.30

SUPPLEMENTARY TABLE 4: Parameters from the equation of state fits for fcc Ni. Here P indicates a paramagnetic calculation, F a ferromagnetic one.

Birch-Murnaghan EOS are shown in Supplementary Fig. 1 f. All other DFT functionals used here fall in between the PW91(FM) and the LSDA+U(FM) curves.

In the large amount of data we produced one can make out different combinations of functional and EOS that fit to the experimentally determined parameters of the textbook case fcc-Ni. It is, however, not our intention here to find the best fit to experiment, but to qualitatively estimate the effects of pressure on the band structure of Ni. Since the data show a relatively small spread we use the average of the curves shown in Supplementary Fig. 1 f as the aggregate "best guess" $P(V)$ curve. We choose the Birch-Murnaghan EOS, but as shown in Supplementary Fig. 1 e, choosing any other EOS would not have altered the results qualitatively. The volumes used in the paper are reported in table 5. We have employed the LDA for the construction of the low-energy $3d + 4s$ orbital models to remain consistent with our ambient pressure calculations.

P (GPa)	100	200	330	500	700	1100
Vol $(\text{a.u.})^3$	54.82	48.23	43.31	39.37	36.25	32.28

SUPPLEMENTARY TABLE 5: The volumes used for the calculations of pressure effects in Ni in the manuscript.

SUPPLEMENTARY NOTE 5. DFT+DMFT SPECTRA

Spectral information can be obtained from CT-QMC data via a transformation of the Green's function from imaginary time or Matsubara axis to real energy. Since this transformation is unstable and the QMC data is inherently noisy, one has to resolve to special techniques, such as maximum entropy [25] or stochastic analytical continuation [26, 27]. Here we use the stochastic optimization method for analytical continuation developed by Mishchenko et al. [28–30] to obtain spectra from the imaginary time Green's function. Since the spectra are not the main focus of this work, we only discuss them here briefly.

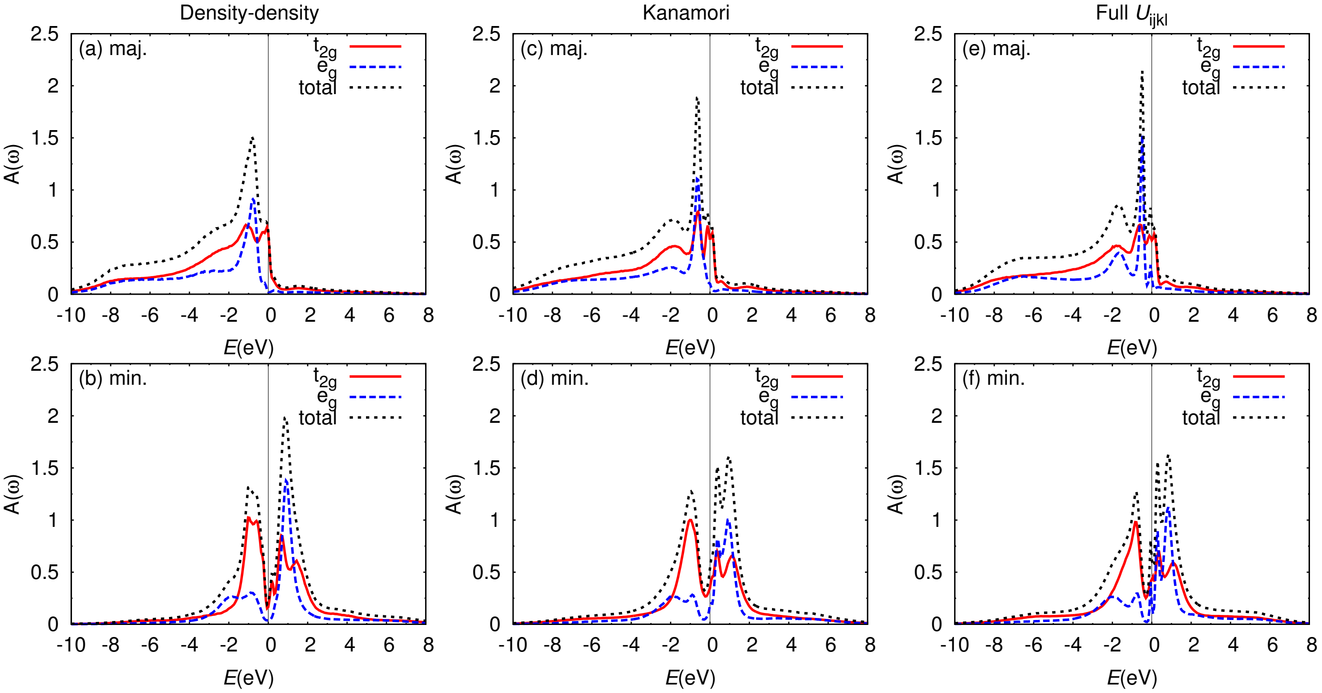

SUPPLEMENTARY FIG. 2: **Iron spectral functions.** Spectra of the $3d$ shell for Fe at $T = 386$K using the three parametrizations of the interaction as discussed above. The top row shows the majority, the bottom row the minority spin channel.

The results for Fe at $T = 386$K are shown in Supplementary Fig. 2 for the three parametrizations of the interaction used. The spectra generally agree well with photoemission experiments [31–33], showing a principal peak below the Fermi level, a secondary peak at about 2eV binding energy, as well as additional features at higher binding energies with a potential satellite at about 6-7eV. The spectra undergo an evolution as a function of the parametrization of the Coulomb interaction, showing more multiplet features when going form density-density (panels a,b in Supplementary Fig. 2) towards the full inclusion of U_{ijkl} (e,f in Supplementary Fig. 2). This is somewhat expected, due to the large effects of the Coulomb interaction on the electronic structure of iron in general, as discussed in the manuscript.

For Ni the situation is complicated by the long history of the satellites in the photoemission spectrum. Our results for Ni ($T = 386$K) are shown in Supplementary Fig. 3 for the three parametrizations of the interaction used. Here, the spectra do not vary as strongly as in the case of iron. This is again not unexpected, since the different parametrizations do not have such a strong effect in Ni. Only small changes in the intensities and positions of features are observed. Comparing to experiments we identify a large principal peak, a sholder next to it, as well as an additional peak at about 2eV binding energy [32, 34–36]. Apart from these, we see only one additional satellite around $6-8$eV binding energy, which we identify as the "6eV valence band satellite". In Supplementary Fig. 4 we show an enlarged version of the satellite region. The whole spectrum below about 4eV binding energy shows stronger majority than minority character, in accordance with experiment [37, 38]. We find the relative spin polarization (majority-spin minus minority-spin divided by their sum) to be between 40% (density-density) and 35% (Kanamori, Full) for the satellite depending on the parametrization of the Coulomb interaction. We do not see additional spectral features beyond the energy regions shown. Satellites reported at higher binding energies have been identified in Ref. [34] as potential artifacts of data subtraction procedures.

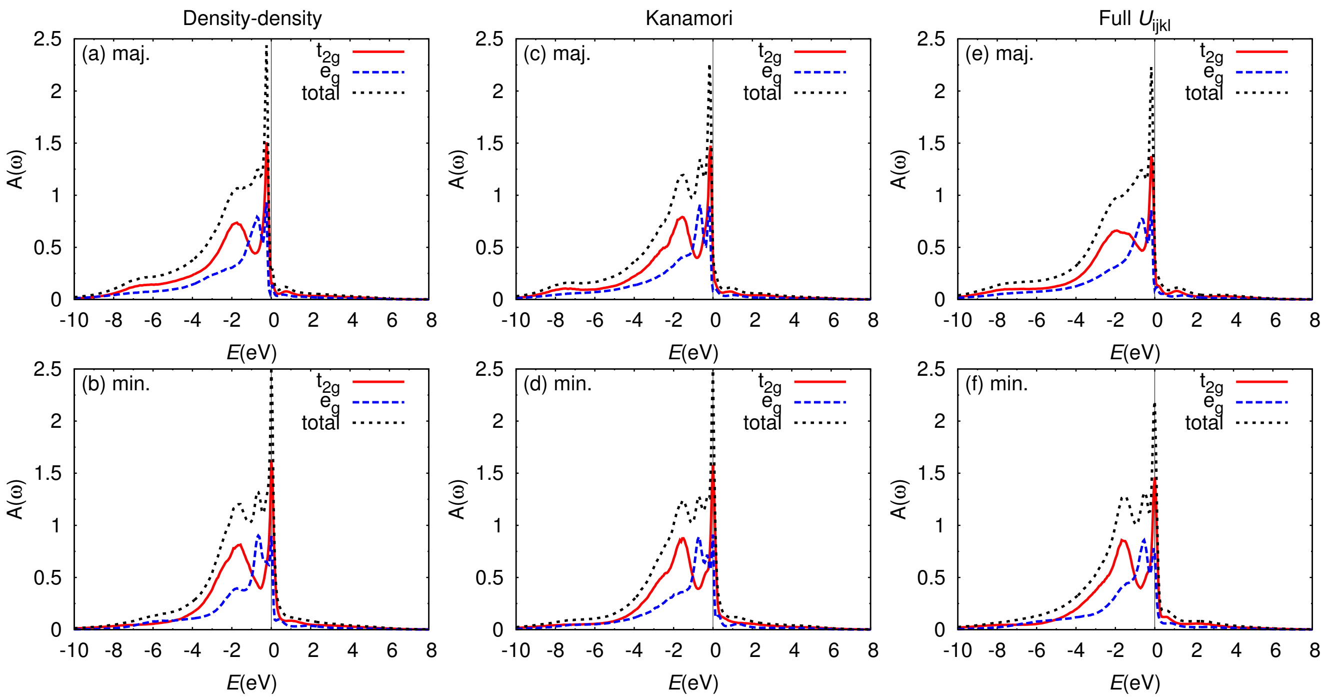

SUPPLEMENTARY FIG. 3: **Nickel spectral functions.** Spectra of the 3d shell for Ni $T = 386$K using the three parametrizations of the interaction as discussed above. The top row shows the majority, the bottom row the minority spin channel.

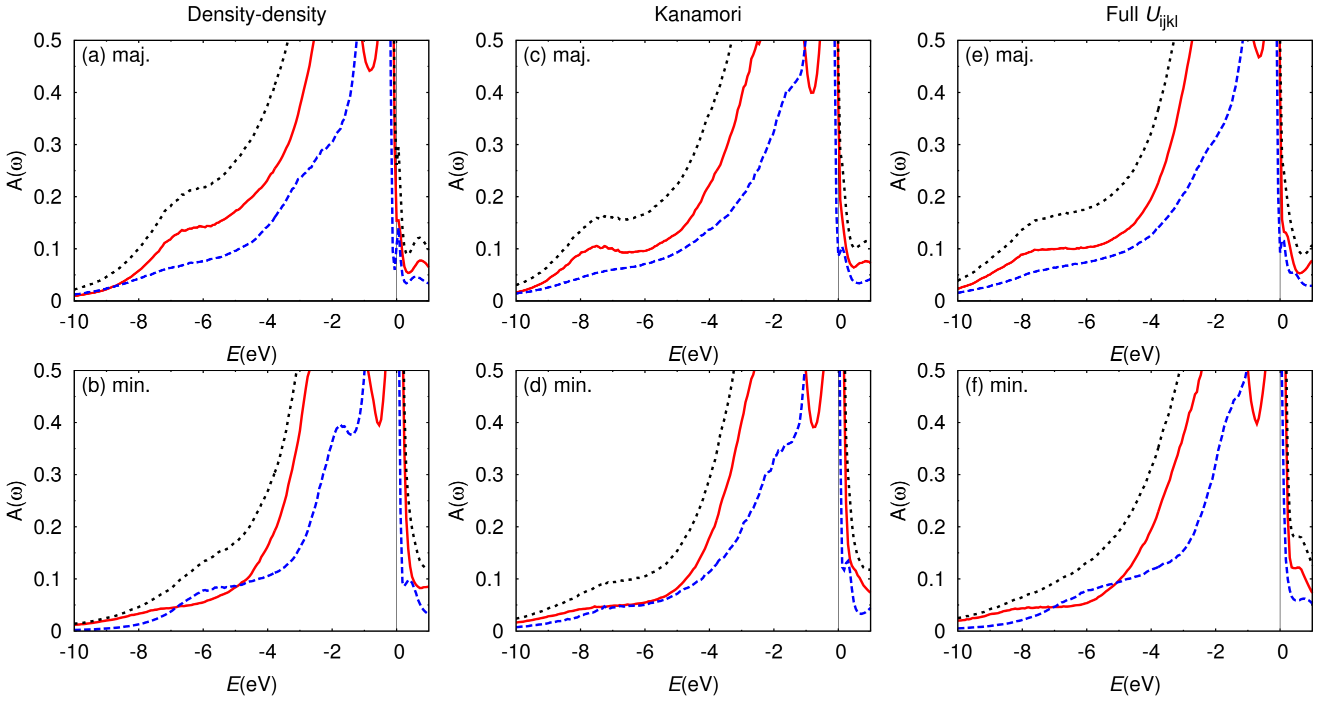

SUPPLEMENTARY FIG. 4: **Ni 6eV-satellite.** Spectra for Ni, with an enlarged satellite region around 6eV. The top row shows the majority, the bottom row the minority spin channel.

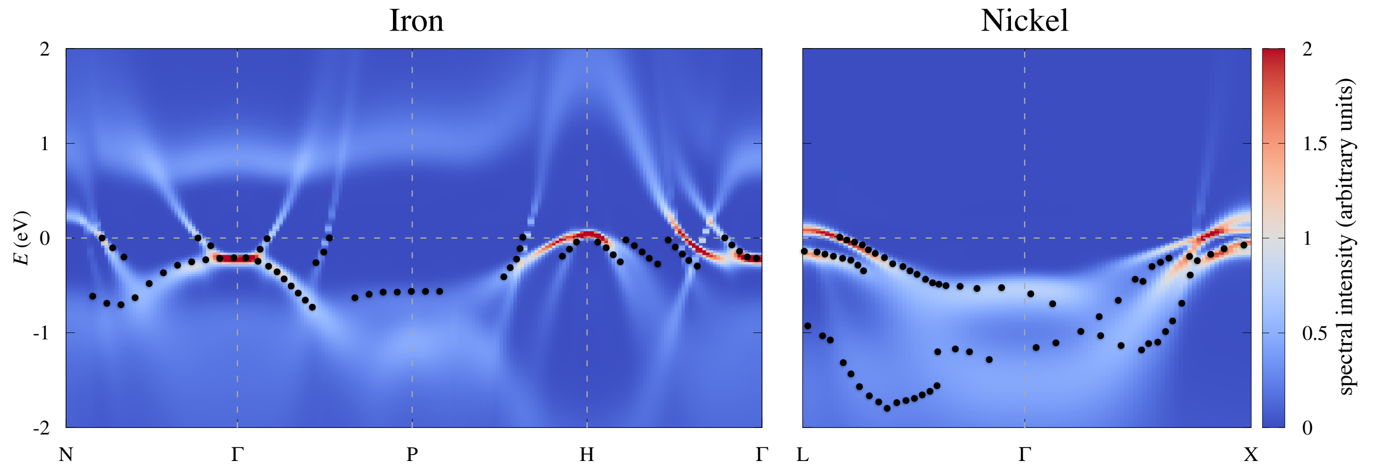

SUPPLEMENTARY FIG. 5: **Momentum resolved spectra.** Total momentum resolved spectral functions of the $3d$ shell for Fe and Ni at $T = 386$K using the density-density parametrization of the interaction as discussed above.

We have also performed a preliminary comparison of our calculations with angle-resolved photoemission spectroscopy (ARPES) data, which is shown in Supplementary Fig. 5. Unlike the local spectral functions shown above, the data were obtained using a faster standard maximum-entropy approach. We compare our results with ARPES of Himpsel et al. [39] for Ni and of Schäfer et al. [40] for Fe. We have used the results of the density-density interaction, since the QMC data at low temperature have the least noise here.

For Ni the agreement is quite satisfactory only close to the Fermi level along L-Γ, but worsens at larger binding energies as well as along Γ-X. From the analytically continued Greens function at the L-point we have estimated the exchange splitting to be about 0.25eV in good agreement with experiments that give values between 0.26 ± 0.05eV [41] and 0.31 ± 0.03eV [39]. Since a recent study [7], employing quasiparticle self-consistent GW (QSGW) to account for non-local correlations, found that using QSGW+DMFT leads to an improved description for the whole band structure, it appears that non-local effects play an important role in ferromagnetic Ni.

For Fe the agreement between our data and ARPES is very good along the N-Γ-P lines of the band structure. Larger quantitative discrepancies appear along P-H-Γ, which is a trend also seen in the Gutzwiller-DFT of Schickling et al. [42]. Since we can reasonably describe the ARPES spectrum of the ferromagnetic phase of Fe, it appears that local correlations play an important role here. On the other hand it was shown in Ref. [7] that the ARPES spectrum of Fe is also very well described within QSGW alone, neglecting local correlations. In this case, only a simultaneous analysis of one- and two-particle quantities within the same theoretical scheme can clarify the relative role of local (DMFT) and non-local (QSGW) correlations.

SUPPLEMENTARY NOTE 6. CPA+DMFT

The coherent potential approximation (CPA) can be combined with DMFT to take into account substitutional disorder, see e.g. Ref. [43] and references therein. Take a binary alloy of two components $A_x B_{1-x}$ with concentrations x and $1-x$.
If the concentration of A is much larger than that of B, one assumes that the dispersion and lattice structure of A is preserved for the whole structure to a good approximation, and that the substituents B are randomly distributed over the lattice sites of A. The substituents B usually have a different number of valence electrons than A. This is accounted for by introducing a level shift ϵ_B at every B site. This shift is estimated from the shift of the static crystal field (center of mass of the density of states) of B as compared to the parent A in a supercell calculation. For the determination of the CPA shifts we used a Fe-hcp supercell with 16 atoms, replacing one of them by Mn or Ni (6.25% admixture).
Since the equation of state of Fe is not well described within DFT, see Supplementary Note 4, following Vekilova et al. we used the volume established within the Preliminary reference Earth model (PREM) [44, 45] to be 7.05Å/atom. The c/a ratio was fixed at 1.6. Since Mn has one $3d$-electron fewer than Fe, and Ni has one $3d$-electron more than Fe, we obtain shifts of $\epsilon_{\mathrm{CPA,Ni}} = -1.67\mathrm{eV}$ for nickel, and $\epsilon_{\mathrm{CPA,Mn}} = +0.94\mathrm{eV}$ for manganese.
Within the DMFT the impurity problem is subsequently solved for each site A and B, and an average is performed over the Greens functions according to their concentrations

$$G_{\mathrm{CPA}} = xG_A(\tau) + (1-x)G_B(\tau). \tag{24}$$

This gives a disorder independent mean-field, that closes the DMFT self-sonsistency loop. We have benchmarked our implementation against Ref. [43] and reproduced some of the results reported there.

SUPPLEMENTARY NOTE 7. MOLECULAR DYNAMICS+DMFT

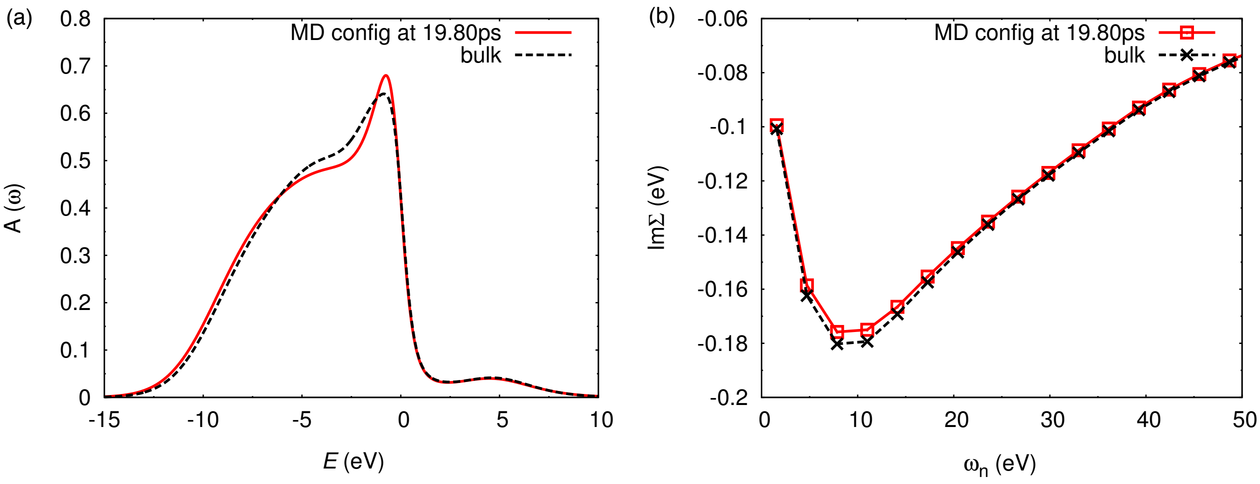

SUPPLEMENTARY FIG. 6: **Molecular Dynamics Simulations.** (a) DMFT spectrum and (b) electronic self-energy averaged over the $3d$ orbitals for the MD configuration at 19.80ps compared to the bulk result obtained using the same parameters.

Since the CPA can account only for substitutional disorder, we have performed additional molecular dynamics (MD) simulations combined with DMFT to estimate effects of thermal disorder. Following Pozzo et al. [46] we used a canonical ensemble at 6000K and a run time of 20 ps and a time step of 1 fs. Configurations were written every 50 fs, so in total 20.000 configurations were generated. These calculations were performed with an fcc Ni supercell at 330 GPa containing 27 atoms. Subsequently, for the last six MD configurations we performed an additional static calculation and a local orbital projection as for the bulk. These inputs were used to perform a DMFT calculation for all 27 Ni atoms in the unit cell. We used a temperature of about 6000K ($\beta = 2\mathrm{eV}^{-1}$) also in the DMFT. Since the crystal structure of the MD configurations is not cubic anymore we used the spherically averaged Coulomb interaction matrix for Ni, see Supplementary Note 1, with $U_{\mathrm{S}} = 2.71\mathrm{eV}$ and $J_{\mathrm{S}} = 1.0\mathrm{eV}$. As expected from the structural changes in each configurations the spectrum, self-energies and fillings vary somewhat between atoms. The fillings of the atoms in each studied configuration vary between 8.8 and 8.9 electrons (bulk: 8.85), where the filling is equally distributed

between the orbitals. The atom-averaged filling in each studied configuration gives 8.84-8.86, basically on top of the bulk result. Averaging the spectra and self-energies over all 27 impurity problems shows that the cell-averaged results are again very close to the bulk fcc Ni result. In Supplementary Fig. 6 we show the DMFT spectrum and electronic self-energy for the MD configuration at 19.80ps compared to the bulk result obtained using the same parameters. The data shown is a goods representative of all MD+DMFT results we obtained for this system.

SUPPLEMENTARY NOTE 8. NONINTERACTING SUSCEPTIBILITY OF IRON

In Supplementary Fig. 7 we show the noninteracting susceptibilty of iron. It has a Curie-Weiss behaviour coming from the e_g orbitals, which have a van-Hove singularity above the Fermi level. Since the DOS is asymmetric around E_{F} and there are minor features, there is also a tiny kink. The t_{2g} part has no sharp feature around E_{F} and thus is of Pauli form.

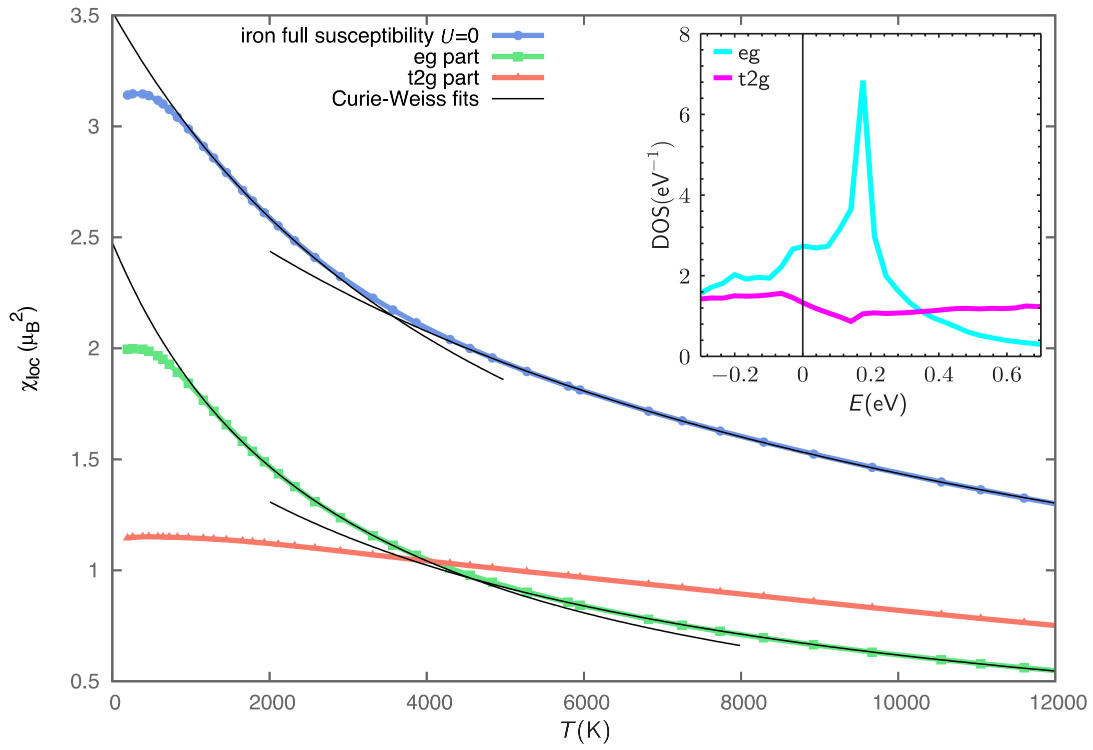

SUPPLEMENTARY FIG. 7: **U=0 Susceptibility of Iron.** The noninteracting susceptibility of iron, as the sum of e_g and t_{2g} contributions, and the noninteracting DOS as inset.

SUPPLEMENTARY NOTE 9. FIRST MATSUBARA RULE

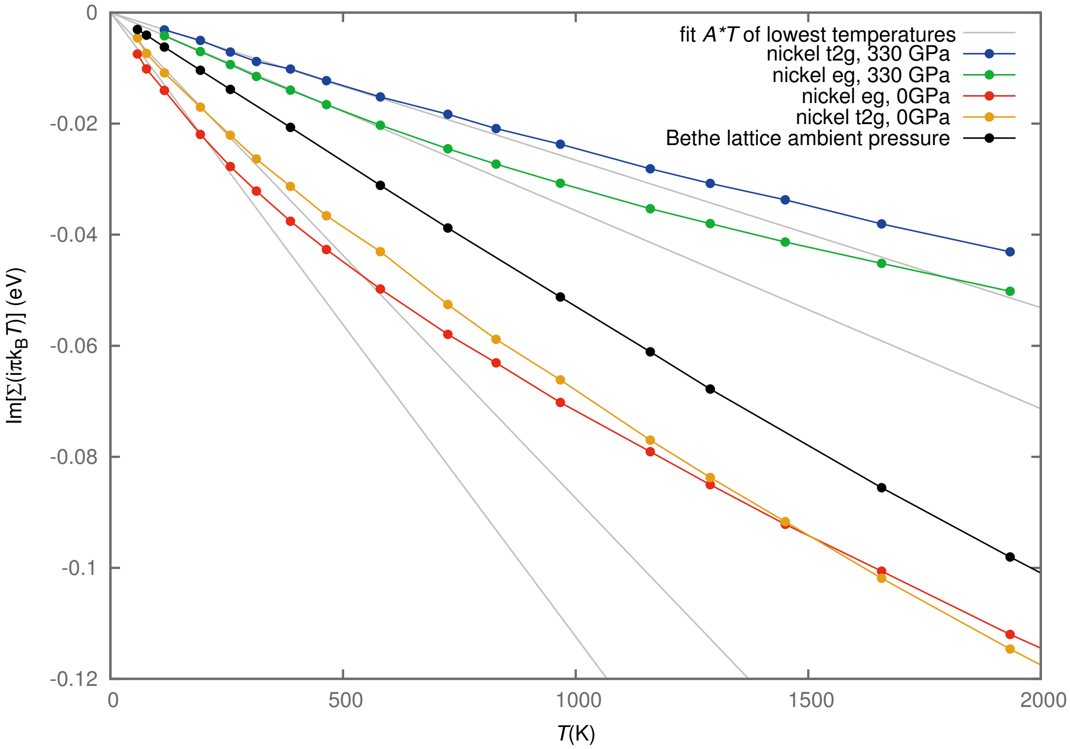

SUPPLEMENTARY FIG. 8: **First Matsubara Rule in Nickel.** For nickel at ambient pressure, at 330GPa and a 3-band Bethe lattice the lowest positive Matsubara frequency of the imaginary part of the self-energy Im[Σ], as well as linear fits of the low-temperature regions.

It is difficult to calculate the scattering rate from data on the imaginary axis. One has either to do an extrapolation of the self-energy or analytic continuation of the data to the real frequency axis. The extrapolation becomes doubtful for large temperatures, since the distance of the first Matsubara frequency $i\pi/\beta = i\pi k_{\mathrm{B}}T$ scales linearly with temperature. Analytic continuation is per construction ill-defined, as discussed above.

One can, however, show, that there is a "first-Matsubara rule" [47], which states that for a canonical Fermi-liquid the scaling of the first Matsubara frequency in temperature has to be $\mathcal{O}(T)$. In Supplementary Fig. 8 we show the result of application of this rule to the 3-band Bethe lattice (90% filled) with a bandwidth of 4.4eV. The result is a line through the origin and thus a Fermi-liquid. Nickel at ambient pressure instead begins to strongly deviate from linear behaviour at a few hundred Kelvins, whereas Nickel at 330GPa pressure begins to deviate at 600K from Fermi-liquid behaviour. This confirms our results for the non-Fermi-liquid scattering rates for nickel shown in the manuscript.

SUPPLEMENTARY NOTE 10. INVERSE SUSCEPTIBILITY OF NI

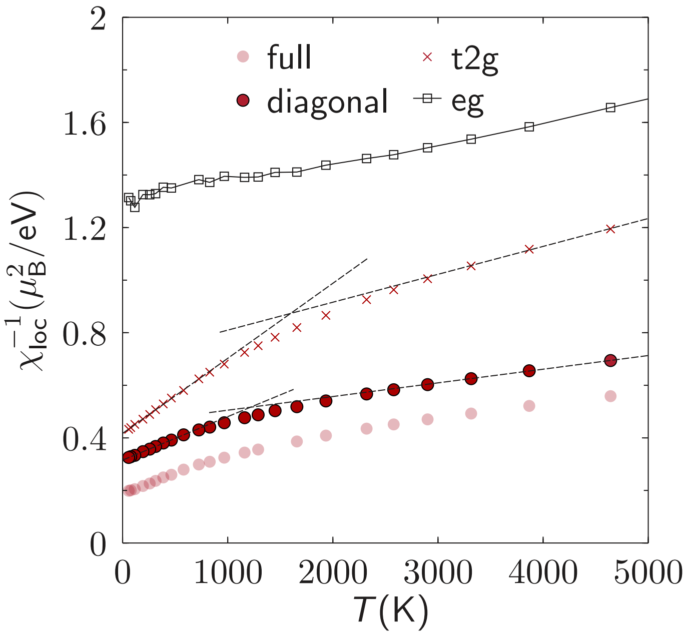

SUPPLEMENTARY FIG. 9: **Inverse Susceptibility of Nickel.** Diagonal, e_g and t_{2g} inverse susceptibility of nickel.

In Supplementary Fig. 9 we show the inverse of the local susceptibility shown in Fig. 2c of the manuscript. The e_g susceptibility, which appears to be flat and thereby Pauli-like in the manuscript is indeed linear in a $1/\chi_{\rm loc}$-plot, since there is a step in the DOS at $E_{\rm F}$. The e_g-bands thus also fulfill a Curie-Weiss law and localize electrons, but have a very large $T_{\rm K}$. The susceptibility only appears Pauli-like in the low-temperature region considered in Fig. 2c, but it is in fact only the tail of the Curie-Weiss function.

SUPPLEMENTARY REFERENCES

[1] Slater, J. C. Quantum theroy of Atomic Structure Vol 1, McGraw-Hill, (1960).

[2] Şaşıoğlu, E., Friedrich, C. & Blügel, S. Effective Coulomb interaction in transition metals from constrained random-phase approximation. *Phys. Rev. B* **83,** 121101(R) (2011)

[3] Kanamori, J. Electron Correlation and Ferromagnetism of Transition Metals *Prog. Theor. Phys.* **30,** 275-289 (1963).

[4] Slater, J. C. The Theory of Complex Spectra. *Phys. Rev.* **34,** 1293-1322 (1929)

[5] Anisimov, V. I., Belozerov, A. S., Poteryaev, A. I. & Leonov, I. Rotationally invariant exchange interaction: The case of paramagnetic iron. *Phys. Rev B* **86,** 035152 (2012)

[6] Antipov, A. E., Krivenko, I. S., Anisimov, V. I., Lichtenstein, A. I. & Rubtsov, A. N., Role of rotational symmetry in the magnetism of a multiorbital model. *Phys. Rev. B* **86,** 155107 (2012)

[7] Sponza, L, Pisanti, P., Vishina, A., Pashov, D., Weber, C., van Schilfgaarde, M., Acharya, S., Vidal, J. & Kotliar, G. Self-energies in itinerant magnets: A focus on Fe and Ni. *Phys. Rev. Lett.* **95,** 041112(R) (2017).

[8] Rohringer, G., Toschi, A., Katanin, A. & Held, K. Critical Properties of the Half-Filled Hubbard Model in Three Dimensions. *Phys. Rev. Lett.* **107,** 256402 (2011)

[9] Karolak, M., Edelmann, M. & Sangiovanni, G. Nickel-titanium double perovskite: A three-dimensional spin-1 Heisenberg antiferromagnet. *Phys. Rev B* **91,** 075108 (2015)

[10] Hirschmeier, D., Hafermann, H., Gull, E., Lichtenstein, A. I. & Antipov, A. E. Mechanisms of finite-temperature magnetism in the three-dimensional Hubbard model. *Phys. Rev B* **92,** 144409 (2015)

[11] Lichtenstein, A. I., Katsnelson, M. I. & Kotliar, G. Finite-Temperature Magnetism of Transition Metals: An *ab initio* Dynamical Mean-Field Theory. *Phys. Rev. Lett.* **87,** 067205 (2001)

[12] Leonov, I., Poteryaev, A. I., Anisimov, V. I. & Vollhardt, D. Electronic Correlations at the α-γ Structural Phase Transition in Paramagnetic Iron. *Phys. Rev. Lett.* **106,** 106405 (2011)

[13] Belozerov, A. S., Leonov, I. & Anisimov, V. I. Magnetism of iron and nickel from rotationally invariant Hirsch-Fye quantum Monte Carlo calculations. *Phys. Rev. B* **87,** 125138 (2013).

[14] Czyżyk, M. T. & Sawatzky, G. A. Local-density functional and on-site correlations: The electronic structure of La_2CuO_4 and $LaCuO_3$. *Phys. Rev. B* **49,** 14211-14228 (1994)

[15] Hewson, A. C. The Kondo Problem to Heavy Fermions. *Cambridge University Press, Cambridge* **34,** (1997)

[16] Karolak, M., Ulm, G., Wehling, T., Mazurenko, V., Poteryaev, A. & Lichtenstein, A. Double counting in LDA+DMFT - The example of NiO. *Journal of Electron Spectroscopy and Related Phenomena*, **181,** 11-15 (2010)

[17] Grånäs, O., Di Marco, I., Thunström, P., Nordström, L., Eriksson, O., Björkman, T., & Wills, J. M. Charge self-consistent dynamical mean-field theory based on the full-potential linear muffin-tin orbital method: Methodology and applications. *Comp. Mat. Sci.* **55,** 295-302 (2012)

[18] Barbiellini, B., Moroni, E.G. & Jarlborg, T. Effects of gradient corrections on electronic structure in metals. *J. Phys.: Condens. Matter.* **2,** 7597 (1990)

[19] Cho, J.-H. & Scheffler, M. *Abinitio* pseudopotential study of Fe, Co, and Ni employing the spin-polarized LAPW approach *Phys. Rev. B* **53,** 10685-10689 (1996)

[20] Mohammed, Y. S., Yan, Y., Wang, H., Li, K. & Du, X. Stability of Ferromagnetism in Fe, Co, and Ni Metals under High Pressure with GGA and $GGA + U$. *Jour. of Magn. and Magn. Mat.* **322,** 653-657 (2010).

[21] Murnaghan, F. D. The Compressibility of Media under Extreme Pressures. *Proc. Natl. Acad. Sci. U.S.A.* **3,** 244-247 (1944)

[22] Birch, F. Finite Elastic Strain of Cubic Crystals. *Phys. Rev.* **71,** 809-824 (1947)

[23] Poirer, J. & Tarantola, A. A logarithmic equation of state. *Phys. Earth. Planet. Inter.* **109,** 1-8 (1998)

[24] Vinet, P., Ferrante, J., Rose, J. H. & Smith, J. R. Compressibility of solids. *J. Geophys. Res.* **92,** 9319-9325 (1987)

[25] Jarrell, M. & Gubernatis, J. E. Bayesian inference and the analytic continuation of imaginary-time quantum Monte Carlo data. *Phys. Rep.* **269,** 133-195 (1996)

[26] Sandvik, A. Stochastic method for analytic continuation of quantum Monte Carlo data. *Phys. Rev. B.* **57,** 10287-10290 (1998)

[27] Beach, K. S. D. Identifying the maximum entropy method as a special limit of stochastic analytic continuation. Preprint at `http://arxiv.org/abs/cond-mat/0403055` (2004).

[28] Mishchenko, A. S., Prokofev, N. V., Sakamoto, A. & Svistunov, B. V. Diagrammatic quantum Monte Carlo study of the Fröhlich polaron. *Phys. Rev. B.* **62,** 6317-6336 (2000)

[29] Mishchenko, A. S., Nagaosa, N., De Filippis, G., de Candia, A. & Cataudella, V. Mobility of Holstein Polaron at Finite Temperature: An Unbiased Approach. *Phys. Rev. Lett.* **114,** 146401 (2015)

[30] Mishchenko, A. S. Stochastic Optimization Method for Analytic Continuation. in *Correlated Electrons: From Models to Materials* , Ch. 14 , FZ Jülich (2012)

[31] Pessa, M., Heimann, P. & Neddermeyer, H. Photoemission and electronic structure of iron. *Phys. Rev. B* **14,** 3488-3493 (1976).

[32] Hüfner, S. & Wertheim, G. K. X-ray photoemission studies of the 3d metals from Mn to Cu. *Phys. Letters* **47A,** 349-350 (1974).

[33] Schulz, A., Courths, R., Schulz, H. & Hüfner, S. UPS investigation of Fe single crystals. *J. Phys. F: Metal Phys.* **9,** L41 (1979).

[34] Nakajima, N., Hatta, S., Odagiri, J., Kato, H. & Sakisaka, Y. Valence-band satellites in Ni: A photoelectron spectroscopic study. *Phys. Rev. B* **70,** 233103 (2004).

[35] Mårtensson, N., Nyholm, R. & Johansson, B. Four-hole satellites in the L_3VV Auger and the valence-band spectra from nickel. *Phys. Rev. B* **30,** 2245-2248 (1984).

[36] Okane, T., Kashiwakura, T., Suzuki, S., Sato, S., Kinoshita, T., Kakizaki, A. & Ishii, T. Angle-resolved resonant photoemission of Ni(100) and Ni(110) single crystals. *Z. Phys. B* **91,** 437-443 (1993).

[37] See, A. K. & Klebanoff, L. E. Spin-Resolved x-ray photoemission from ferromagnetic nickel. *Phys. Rev. B* **51,** 11002-11011 (1995).

[38] Kakizaki, A., Ono, K., Tanaka, K., Shimada, K. & Sendohda, T. Spin-Resolved photoemission of valence-band satallites in Ni. *Phys. Rev. B* **55,** 6678-6681 (1997).

[39] Himpsel, F. J., Knapp, J. A. & Eastman, D. E. Experimental energy-band dispersions and exchange splitting for Ni. *Phys. Rev. B* **19,** 2919-2927 (1979).

[40] Schäfer, J., Hoinkis, M., Rotenberg, E., Blaha, P. & Claessen, R. Fermi surface and electron correlation effects of ferromagnetic iron. *Phys. Rev. B* **72,** 155115 (2005).

[41] Eberhardt, W. & Plummer, E. W. Angle-resolved photoemission determination of the band structure and multielectron excitations in Ni. *Phys. Rev. B* **21,** 3245-3255 (1980).

[42] Schickling, T., Bünemann, J., Gebhard, F. & Boeri, L. Quasiparticle bands and structural phase transition of iron from Gutzwiller density-functional theory. *Phys. Rev. B* **93,** 205151 (2016).

[43] Poteryaev, A. I., Skornyakov, S. L., Belozerov, A. S. & Anisimov, V. I. Specific heat of a binary alloy within the CPA+DMFT method. *Phys. Rev. B* **91,** 195141 (2015).

[44] Dziewonski, A. N. & Anderson, D. L. Preliminary reference Earth model. *Phys. Earth Planet. Inter.* **25**, 297-356 (1981).

[45] Vekilova, O. Y., Pourovskii, L. V., Abrikosov, I. A. & Simak, S. I. Electronic correlations in Fe at Earth's inner core conditions: Effects of alloying with Ni. *Phys. Rev. B* **91,** 245116 (2015).

[46] Pozzo, M., Davies C., Gubbins, D. & Alfè, D. Thermal and electrical conductivity of iron at Earths core conditions. *Nature* **485,** 355358 (2012).

[47] Chubukov, A. V. & Maslov, D. L. First-Matsubara-Frequency rule in a Fremi liquid. I. Fermionic self-energy. *Phys. Rev. B* **86,** 155136 (2012).
